# Sensing Technologies in Industrial Defect Detection

**DOI:** 10.3390/s26185706

**Published:** 2026-09-09

**Authors:** Peng Liu

**Affiliations:** 1 Key Laboratory of CNC Equipment Reliability, Ministry of Education, Jilin University, Changchun 130025, China; liupeng@jlu.edu.cn; 2 School of Mechanical and Aerospace Engineering, Jilin University, Changchun 130022, China

## 1. Introduction

Industrial defect detection is an essential component of modern manufacturing and plays a critical role in ensuring product quality, operational reliability, and production safety. With the continuing development of intelligent manufacturing, industrial inspection is evolving from conventional offline and operator-dependent examination toward automated, continuous, and data-driven sensing. Machine vision has become one of the most widely adopted approaches for surface and appearance inspection, while non-destructive evaluation techniques such as ultrasonic testing and infrared thermography provide complementary capabilities for detecting internal or subsurface defects [1,2,3]. These sensing technologies have substantially expanded the observability of manufacturing processes and engineering components, enabling defects to be detected, localized, and characterized with increasing spatial, temporal, and quantitative resolution.

At the same time, advances in sensor networks, data acquisition, signal processing, and artificial intelligence are extending industrial sensing beyond isolated defect identification. Multi-sensor data fusion allows complementary information from different sensing modalities to be integrated for more reliable condition assessment and fault diagnosis [4]. In parallel, prognostics and health management has established a broader framework in which sensing data are used not only to detect abnormal conditions but also to diagnose degradation, predict future system states, and support condition-based or predictive maintenance [5]. Nevertheless, the transition from laboratory demonstrations to reliable industrial deployment remains challenging. Variations in operating conditions and data distributions can substantially reduce the generalization performance of data-driven diagnostic models [6]. The increasing use of deep learning also raises concerns regarding model transparency and the physical credibility of diagnostic decisions, particularly in safety- and quality-critical applications [7]. For prognostic tasks, uncertainty, robustness, interpretability, and practical feasibility are increasingly recognized as important requirements in addition to prediction accuracy alone [8].

Against this background, the Special Issue “Sensing Technologies in Industrial Defect Detection” was established to present recent developments in sensing, inspection, condition monitoring, fault diagnosis, prognosis, and intelligent maintenance for industrial applications. The contributions collected in this Special Issue span multiple sensing modalities and analytical paradigms, including machine vision, infrared thermography, X-ray computed tomography, vibration and multi-source sensing, process and equipment monitoring, machine learning, uncertainty-aware prediction, and intelligent maintenance decision making. Although these studies address different industrial systems and defect or fault scenarios, they collectively reflect a common evolution in the field: industrial sensing is progressing from the acquisition of defect-related signals toward integrated frameworks capable of extracting physically meaningful information, evaluating equipment or product condition, predicting degradation, and supporting operational decisions. This Special Issue therefore provides a timely perspective on the increasingly close integration of sensing technologies, signal and image processing, artificial intelligence, reliability assessment, and intelligent manufacturing.

## 2. Overview of Published Papers

Several studies focus directly on defect and quality inspection. Solovev et al. (6) present an infrared-thermography-based framework for in-process laser welding monitoring, in which spatter and weld-zone thermal features are combined with machine learning to identify multiple welding defects. Saiyod et al. (4) address printed circuit board inspection using a training-free, reference-based image-processing framework, highlighting the continued value of interpretable deterministic methods when labeled data are limited or auditability is required. Chang et al. (10) propose an explainable multimodal neural network for final-state quality inspection of hairpin windings, combining image information with span-related geometric priors. These studies demonstrate that industrial defect detection increasingly benefits from the integration of physical or geometric knowledge with data-driven models rather than relying solely on generic image classifiers.

Other papers extend the Special Issue from direct defect localization to degradation assessment and condition monitoring. Chai et al. (1) develop a hierarchical multi-scale temporal convolutional network for tool condition monitoring using multi-sensor signals, while Zhang et al. (3) propose an adaptive signal-decomposition and clustering strategy for bearing fault detection. Both studies emphasize the importance of extracting weak, multi-scale, and temporally evolving features from complex sensor signals. Zhang et al. (2) link mesoscopic defect characteristics obtained through X-ray computed tomography to fatigue life in 6061-T6 aluminum alloy, illustrating how defect sensing can support quantitative assessment of subsequent structural performance.

The Special Issue also includes studies that move beyond diagnosis toward prognosis and operational decision making. Qu et al. (7) address remaining useful life prediction through Bayesian convolutional neural networks with dual-output units, explicitly considering aleatoric and epistemic uncertainty. Dai et al. (8) propose a two-stage wind turbine fault detection strategy for highly imbalanced SCADA data, combining anomaly screening with supervised classification. Liu et al. (9) further connect state information with maintenance planning through a multi-head deep reinforcement learning approach for joint maintenance decisions in an electro-hydraulic servo fatigue testing machine. At the system level, Liu et al. (5) integrate wearable sensing and multi-source information with enhanced Petri nets to improve production system monitoring and real-time state representation.

Taken together, these studies show that the scope of industrial defect detection is becoming broader and more interconnected. Nevertheless, several challenges remain. First, sensing performance under variable industrial conditions, including illumination changes, environmental interference, operating-condition drift, sensor noise, and domain shifts, still limits model transferability. Second, high-quality labeled defect data are often scarce, imbalanced, or expensive to obtain, particularly for rare but safety-critical defects. Third, multimodal sensing can improve observability but introduces new issues in calibration, synchronization, feature alignment, and information redundancy. Fourth, increasingly complex learning models require stronger interpretability, uncertainty quantification, and traceable decision mechanisms before they can be trusted in safety- or quality-critical production environments.

Future research should therefore place greater emphasis on physics-informed and knowledge-guided learning, robust multimodal fusion, self-supervised and few-shot learning, uncertainty-aware diagnosis, and explainable artificial intelligence. Equally important is the transition from laboratory demonstrations to long-term industrial validation. Sensor systems and algorithms should be evaluated across machines, materials, production batches, and operating conditions using standardized metrics and reproducible protocols. Edge computing and lightweight models will also be important for real-time deployment, while digital twins and closed-loop manufacturing systems may provide a pathway for converting sensing results into adaptive process control and maintenance actions.

## 3. Conclusions

The papers collected in this Special Issue demonstrate the continuing evolution of industrial sensing from defect observation toward intelligent condition understanding. The contributions cover multiple sensing modalities and analytical paradigms, ranging from deterministic image comparison and thermal monitoring to multi-sensor temporal learning, uncertainty-aware prognosis, and reinforcement-learning-based maintenance decisions. Their diversity reflects the fact that no single sensor or algorithm is universally optimal for industrial defect detection; successful solutions depend on the physical characteristics of the target defect, the manufacturing environment, data availability, and the required level of interpretability and real-time performance.

Looking ahead, progress will increasingly depend on the coordinated development of sensing hardware, data analytics, domain knowledge, and industrial deployment strategies. Robustness, generalization, data efficiency, multimodal integration, uncertainty quantification, and explainability remain central research priorities. By addressing these challenges, sensing technologies can support a transition from reactive inspection toward continuous, predictive, and eventually adaptive quality assurance. We hope that the contributions in this Special Issue will provide useful references for researchers and engineers and stimulate further interdisciplinary research in intelligent industrial inspection and smart manufacturing.
